# Added Value of [18F]PSMA‐1007 PET/CT and PET/MRI in Patients With Biochemically Recurrent Prostate Cancer: Impact on Detection Rates and Clinical Management

**DOI:** 10.1002/jmri.29386

**Published:** 2024-04-28

**Authors:** Bendik S. Abrahamsen, Torgrim Tandstad, Bjørg Y. Aksnessæther, Trond V. Bogsrud, Miguel Castillejo, Eivor Hernes, Håkon Johansen, Thomas M. I. Keil, Ingerid S. Knudtsen, Sverre Langørgen, Kirsten M. Selnæs, Tone F. Bathen, Mattijs Elschot

**Affiliations:** ^1^ Department of Circulation and Medical Imaging Norwegian University of Science and Technology Trondheim Norway; ^2^ The Cancer Clinic, St. Olavs Hospital Trondheim University Hospital Trondheim Norway; ^3^ Department of Clinical and Molecular Medicine Norwegian University of Science and Technology Trondheim Norway; ^4^ Department of Oncology Ålesund Hospital, Møre and Romsdal Hospital Trust Ålesund Norway; ^5^ PET Imaging Centre University Hospital of North Norway Tromsø Norway; ^6^ PET‐Centre Aarhus University Hospital Aarhus Denmark; ^7^ Division of Radiology and Nuclear Medicine Oslo University Hospital Oslo Norway; ^8^ Department of Radiology and Nuclear Medicine St. Olavs Hospital, Trondheim University Hospital Trondheim Norway

**Keywords:** PET/MRI, PET/CT, prostate cancer, biochemical recurrence, PSMA PET

## Abstract

**Background:**

Prostate‐specific membrane antigen (PSMA) positron emission tomography (PET) can change management in a large fraction of patients with biochemically recurrent prostate cancer (BCR).

**Purpose:**

To investigate the added value of PET to MRI and CT for this patient group, and to explore whether the choice of the PET paired modality (PET/MRI vs. PET/CT) impacts detection rates and clinical management.

**Study Type:**

Retrospective.

**Subjects:**

41 patients with BCR (median age [range]: 68 [55–78]).

**Field Strength/Sequence:**

3T, including T1‐weighted gradient echo (GRE), T2‐weighted turbo spin echo (TSE) and dynamic contrast‐enhanced GRE sequences, diffusion‐weighted echo‐planar imaging, and a T1‐weighted TSE spine sequence. In addition to MRI, [^18^F]PSMA‐1007 PET and low‐dose CT were acquired on the same day.

**Assessment:**

Images were reported using a five‐point Likert scale by two teams each consisting of a radiologist and a nuclear medicine physician. The radiologist performed a reading using CT and MRI data and a joint reading between radiologist and nuclear medicine physician was performed using MRI, CT, and PET from either PET/MRI or PET/CT.

Findings were presented to an oncologist to create intended treatment plans. Intrareader and interreader agreement analysis was performed.

**Statistical Tests:**

McNemar test, Cohen's *κ*, and intraclass correlation coefficients. A *P*‐value <0.05 was considered significant.

**Results:**

7 patients had positive findings on MRI and CT, 22 patients on joint reading with PET/CT, and 18 patients joint reading with PET/MRI. For overall positivity, interreader agreement was poor for MR and CT (*κ* = 0.36) and almost perfect with addition of PET (PET/CT *κ* = 0.85, PET/MRI *κ* = 0.85). The addition of PET from PET/CT and PET/MRI changed intended treatment in 20 and 18 patients, respectively. Between joint readings, intended treatment was different for eight patients.

**Data Conclusion:**

The addition of [^18^F]PSMA‐1007 PET/MRI or PET/CT to MRI and CT may increase detection rates, could reduce interreader variability, and may change intended treatment in half of patients with BCR.

**Level of Evidence:**

3

**Technical Efficacy:**

Stage 3

Rising serum prostate‐specific antigen (PSA) values after prostate cancer treatment with curative intent indicate presence of recurrent or persistent disease. This condition, known as biochemical recurrent prostate cancer (BCR), eventually occurs in more than 25% of patients after definitive local therapy.^1^, ^2^, ^3^

^18^F‐ and ^68^Ga‐prostate‐specific membrane antigen (PSMA)‐radioligand positron emission tomography (PET) imaging has shown to have a considerable impact on treatment for this group of patients.^4^, ^5^, ^6^, ^7^, ^8^ When compared with conventional imaging, changes in intended treatment are reported in as many as 50% of the patients.^4^, ^5^, ^6^, ^7^, ^8^ In selected centers, PET/MRI examinations are an alternative to the more commonly available PET/computed tomography (CT) examinations. In recent studies comparing the detection rates of prostate cancer recurrence between [^68^Ga]Ga‐PSMA‐11 PET/MRI and [^68^Ga]Ga‐PSMA‐11 PET/CT, PET/MRI was found to detect lymph node metastases and distant metastases at least as well as PET/CT.^9^, ^10^ Furthermore, it has been reported to be superior in detecting local recurrences.^11^, ^12^ Additionally, MRI has been shown to outperform CT for the detection of vertebral metastases,^13^ indicating that MRI may be a better anatomical correlate for PET than CT for localizing bone metastases.

For imaging, [^18^F]PSMA‐1007 is an alternative PET tracer to [^68^Ga]Ga‐PSMA‐11.^14^ Specifically, [^18^F]PSMA‐1007 has primarily hepatobiliary excretion and low urinary excretion.^14^ This is favorable for detection of local recurrences compared with [^68^Ga]Ga‐PSMA‐11, which is excreted primarily through the urinary system.^14^, ^15^

Against this background, the aim of this study was to examine the impact of adding [^18^F]PSMA‐1007 PET to MRI and CT regarding localizing recurrent disease and to investigate the impact on further clinical management, and to compare whether the origin of the PET signal (i.e., whether PET was from PET/MRI or PET/CT) may have an impact on the detection rates and clinical management of patients with BCR.

## Materials and Methods

This study is a retrospective analysis of prospectively acquired data obtained with passive informed consent (opt‐out) that was approved by the regional research ethics committee (REK 2014/1289).

### Study Population

The study population consisted of a cohort of 46 patients with BCR after primary treatment for prostate cancer or residual disease after salvage treatment. Specifically, BCR was defined according to the European Association of Urology—European Society for Radiotherapy and Oncology—International Society of Geriatric Oncology Guidelines on Prostate Cancer.^3^ All patients had PET/CT and PET/MRI available that was performed on the same day and after a single injection with [18F]PSMA‐1007.

Images from 5 of the 46 patients were used to calibrate the reading protocol between readers before the study start. The remaining 41 patients were included in the analysis. Of these patients, 25 (61%) were referred owing to BCR after initial treatment and 16 (39%) were referred owing to suspicion of residual disease after salvage therapy. Patient characteristics can be found in Table 1.

**TABLE 1:** Patient Characteristics at the Time of Imaging for the 41 Patients Included in the Analysis

Clinical variable	Value
Age (years)	68 [55–78]
PSA value (ng/mL)	0.80 [0.20–82.62]
Indication for imaging	
BCR after initial treatment	25 (61%)
Recurrence after salvage treatment	16 (39%)
ISUP grade group at primary diagnosis	
GG 2	13 (32%)
GG 3	13 (32%)
GG 4	8 (20%)
GG 5	5 (12%)
Unknown	2 (5%)
Primary treatment	
RP	22 (54%)
RP + PLND	15 (37%)
RT	4 (10%)
Salvage treatment	
No salvage treatment	25 (61%)
Salvage RT	
Prostate bed	7 (17%)
Prostate bed and lymph nodes	6 (15%)
Salvage PLND	2 (5%)
Salvage prostatectomy	1 (2%)
Imaging and post‐injection times	
WB and pelvic PET (n)	21 (51%)
PET/CT PI WB (minutes)	120 [114–153]
PET/MRI PI WB (minutes)	175 [22–254]
PET/MRI PI Pelvic (minutes)	208 [56–288]
WB PET only (n)	9 (22%)
PET/CT PI (minutes)	120 [120–143]
PET/MRI PI (minutes)	182 [95–213]
Pelvic PET only (n)	11 (27%)
PET/CT PI (minutes)	147 [125–182]
PET/MR PI (minutes)	142 [49–214]

PSA = prostate‐specific antigen; BCR = biochemically recurrent prostate cancer; ISUP = The International Society of Urological Pathology; GG = grade group; RP = radical prostatectomy; PLND = pelvic lymph node dissection; RT = radiotherapy; WB = whole‐body; PET = positron emission tomography; PI = post‐injection time; CT = computed tomography.

### Study Design, Image Evaluation, and Intention‐to‐Treat Analysis

**FIGURE 1:**
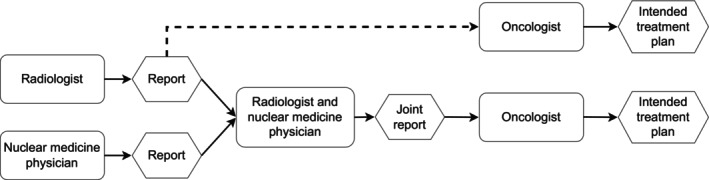
Study design. PET and low‐dose CT images were read by nuclear medicine physicians. MRI and CT images were read by radiologists. A joint reading was performed between radiologist and nuclear medicine physician using all available image data. Joint readings and radiologist readings formed the basis for an intention‐to‐treat analysis performed by a urological oncologist.

The study design is summarized in Fig. 1. The images were reported by two reading teams from different centers referred to as the primary and secondary reading team. Each reading team consisted of one radiologist and one nuclear medicine physician. The primary reading team consisted of a nuclear medicine physician with 10 years of experience (EH) and a radiologist with 25 years of experience (SL). The secondary reading team consisted of a nuclear medicine physician with 24 years of experience (TVB) and a radiologist with 30 years of experience (MC).

Within each team, multiple sets of readings were performed: 1) The nuclear medicine physician read PET images where low‐dose axial CT images were used as anatomical correlate. The readings were performed in two folds. Within each fold, half of the patients were represented by PET from PET/MRI (PET_PET/MRI_) and half with PET from PET/CT (PET_PET/CT_). For the PET_PET/MRI_ images, the low‐dose CT was coregistered into the MRI space. Each fold was read at least two weeks apart. 2) Reading of the MRI and CT images was performed by the radiologist. As only the PET data differed between folds, the radiologist read a single fold only. 3) Joint readings were performed between the radiologist and the nuclear medicine physician in each reading team, where their individual readings formed the basis of the joint reading. Like the nuclear medicine physician's readings, the joint readings were performed in two folds where each fold had half of the patients with PET_PET/MRI_ images and half with PET_PET/CT_ images. PET/CT + MRI and PET/MRI + CT are in the following used to refer to the joint readings with PET_PET/CT_ and PET_PET/MRI_, respectively. Both MRI and CT data were also available to the readers during the joint readings. MRI + CT is used to describe the radiologist readings where only MRI and CT images were available.

Finally, an intention‐to‐treat analysis was performed by a urological oncologist with 20 years of experience (TT) based on the results from the radiologist readings and from both joint readings (i.e., six treatment plans were created for each patient, three from each reading team). The data for the joint readings were presented in the same folds as used by the readers during image interpretation, where half of the patients were represented by PET_PET/MRI_ and half with PET_PET/CT_. Each dataset was presented for the urological oncologist at least one week apart. In addition to lesion findings and interpretations, relevant clinical information such as prior treatment history, International Society of Urological Pathology grade group, and PSA history was provided.

The treatment strategies were based on a synthesis of the European Association of Urology guidelines for prostate cancer^16^ and the official Norwegian guidelines for prostate cancer.^17^ Intended treatment was categorized into androgen deprivation therapy (ADT) with confirmed metastatic disease (ADT M1), ADT without confirmed metastatic disease (ADT M0), salvage radiotherapy, metastasis‐directed therapy, observation, and further examinations. The latter category covered cases where additional workup would have been required prior to determining further treatment, such as biopsies of suspicious lesions or bone scintigraphy in cases of possible bone metastasis outside the PET field of view (FOV). When comparing change in clinical management between datasets, further examinations were only considered equal if the suggested examinations were the same (eg, biopsy of the same lesion). When comparing the aggressiveness of the clinical management between datasets the intended treatments were ordered from least to most aggressive as follows: observation, salvage radiotherapy, metastasis‐directed therapy, ADT M0, and ADT M1.

To evaluate the variability in intended treatment plans among oncologists, a second urologic oncologist (BYA) with 14 years of expertise conducted an intention‐to‐treat analysis. This analysis was based on the radiologist and joint reading data from the primary reading team and adhered to the same protocol employed in the initial intention‐to‐treat analysis.

### Imaging Protocols and Processing

The PET_PET/CT_ and CT images were acquired on a Siemens Biograph mCT PET/CT scanner (Siemens Healthineers, Erlangen, Germany). The images were acquired from skull base to proximal thighs or vertex to proximal thighs with 3.0–3.5 minutes per bed position. The images were reconstructed using ordered subset expectation maximization (2 iterations and 21 subsets) with point‐spread function modeling, time‐of‐flight, and a 4‐mm Gaussian post‐reconstruction smoothing filter. The reconstruction was done on a 256 × 256 matrix using pixel spacing 3.2 × 3.2 mm^2^ and a slice thickness of 3 mm. Model‐based scatter scaling was used for PET_PET/CT_ reconstructions.^18^

The acquired CT images were low‐dose images acquired without intravenous contrast. The low‐dose CT images were used for attenuation correction of the PET_PET/CT_ images and for anatomical correlation. The images were acquired with a tube voltage of 120 kV and adaptive exposure control with tube current values ranging from 23 to 173 mA. Using filtered back‐projection, images were reconstructed onto a 512 × 512 matrix with an in‐plane resolution of 0.98 × 0.98 mm^2^ and a slice thickness of 3 mm.

The PET_PET/MRI_ and MRI data were acquired on a 3‐T Siemens Biograph mMR PET/MRI scanner (Siemens Healthineers, Erlangen, Germany). The whole‐body PET_PET/MRI_ images were acquired from skull base to proximal thighs using four bed positions with 4–6 minutes per bed position. The images were reconstructed using ordered subset expectation maximization (3 iterations and 21 subsets) with point‐spread function modeling and a 4‐mm Gaussian post‐reconstruction smoothing filter. The reconstruction was done on a 344 × 344 matrix using pixel spacing 2.1 × 2.1 mm^2^ with a slice thickness of 2 mm. The PET_PET/MRI_ pelvic series had a single 6–10 minutes bed position with otherwise equal reconstruction parameters. Attenuation correction was performed using the default five‐compartment MRI‐based attenuation correction wherein Dixon MRI was segmented into tissue classes air, soft tissue, fat and lung, and bone information was added using an atlas‐based approach.^19^ Model‐based scatter scaling was used for PET_PET/MRI_ reconstructions.^18^

The MRI protocol included pelvic sequences to evaluate local recurrences, regional stage and pelvic bone metastases and whole‐body sequences to evaluate distant metastases. The pelvic MRI protocol included a high‐resolution three‐dimensional T2‐weighted (T2w) sampling perfection with application‐optimized contrasts using different flip angle evolution (SPACE) sequence, diffusion‐weighted imaging (DWI, b50‐b800), and T1‐weighted (T1w) dynamic contrast‐enhanced (DCE) images. For 21 patients, an additional high‐resolution pelvic DWI sequence was acquired. The whole‐body protocol included a T1w Dixon sequence, a T2w half‐Fourier acquisition single‐shot turbo spin echo (HASTE) sequence, and a T1w TSE sequence covering the spine. For one patient, DCE‐MRI and high‐resolution pelvic T2w images were not available. Detailed information about the MRI protocol can be found in Table 2.

**TABLE 2:** Parameters for Acquired MRI Sequences

Sequence	Base Sequence	Orientation	Slice Thickness (mm)	TE (msec)	TR (msec)	Flip Angle	Acquisition Matrix	In‐plane Resolution (mm)	*n*	Additional Information
T2 SPACE	TSE	Axial	1.0	90	1300	110°	640 × 640	0.5 × 0.5	40	Pelvic FOV
T1 Dixon VIBE	GRE	Coronal	3.0	2.6	4.2	9°	737 × 261	1.4 × 1.4	39	
T1 DCE VIBE	GRE	Axial	3.3	1.2	5.6	10°	320 × 320	0.7 × 0.7	40	Pelvic FOV, Clariscan™ body weight‐adapted
DWI (b50, b800) and ADC	GRE	Axial	4.4	54	6600	90°	180 × 203	2.0 × 2.0	39	Pelvic FOV
T1	TSE	Sagital	4.0	9.5	500	140°	974 × 387	1.0 × 1.0	39	FOV covering spine
T2 HASTE	TSE	Axial	4.0	96	1600	120°	320 × 320	1.2 × 1.2	39	Whole body
DWI (b50, b800) and ADC (small FOV)	GRE	Axial	3.0	63	4600	90°	256 × 256	1.1 × 1.1	21	Pelvic FOV

Acquisition parameters were in some cases adapted for patient comfort concerns or owing to scanning schedule.

TR = repetition time; TE = echo time; *n* = number of patients for which the series was acquired; SPACE = sampling perfection with application optimized contrasts using different flip angle evolution; TSE = turbo spin echo; FOV = field of view; GRE = gradient echo; VIBE = volume interpolated GRE; HASTE = half‐Fourier acquisition single‐shot TSE; DWI = diffusion‐weighted imaging; DCE = dynamic contrast‐enhanced.

The imaging data were deidentified and split into folds such that each fold contained approximately the same number of patient examinations from each PET modality. The readers were blinded to whether a given PET image originated from PET/MRI or PET/CT and were only given access to the images relevant for the current reading. In cases where only a pelvic FOV was acquired for the PET_PET/MRI_ images, the PET_PET/CT_ images were cropped to a similar FOV to reduce the chances of finding lesions in PET_PET/CT_ that would be outside the PET_PET/MRI_ FOV. Additionally, 2.5 cm was cropped from the pelvic FOV owing to the reduced sensitivity at the axial extremes of the PET acquisition.^20^

Registration of the low‐dose CT images to the MRI space was used as an anatomical correlate during the nuclear medicine physician reading of the PET_PET/MRI_ data. The registration was performed using a rigid registration scheme.^21^, ^22^

### Image Interpretation

In all readings, each lesion was scored on a five‐point Likert scale analogous with European Association of Nuclear Medicine reader's confidence^23^: 1—benign; 2—most likely benign; 3—equivocal; 4—most likely malignant; 5—malignant. The readers were provided with a limited subset of clinical patient information, including the PSA level at scan time and information about the primary curative treatment. The images were read in syngo.via (Siemens Healthineers, Erlangen, Germany) or Sectra PACS (Sectra, Linköping, Sweden).

### Detection Rate and Intrareader and Interreader Agreement Analysis

A patient‐level and region‐based detection rate analysis was performed. A patient or region was considered positive if it contained one or more positive lesions. Findings with scores 4 and 5 were considered positive. Lesions scored lower were considered benign and omitted from further analysis.

An interreader (between the reading teams) and intrareader (between imaging modalities within each reading team) agreement analysis was performed for overall patient positivity and disease stage. In the disease stage, analysis recorded findings were used to stage patients according to the molecular imaging TNM (miTNM) system.^24^ According to the miTNM system, the presence of lymph node metastases in a single pelvic lymph node region (internal/external/common iliac, obturator, presacral, and other pelvic) is reported as miN1 and presence of metastases in two or more pelvic lymph node regions is described as miN2. Presence of lymph node metastases outside the pelvis is reported as miM1a, presence of bone metastasis as miM1b and presence of visceral organ metastasis as miM1c. The evaluation of local recurrence was modified from the miTNM system, and a binary classification system (miT0/miT+) was used for all patients, regardless of whether the patient had undergone radical prostatectomy or radiotherapy as primary curative treatment.

### Statistical Analysis

The statistical analysis was performed in Python using statsmodels (version 0.10.0, https://www.statsmodels.org/)^25^ to perform the McNemar test and calculate Cohen's *κ* and pingouin (version 0.3.3, https://pingouin-stats.org/)^26^ to calculate the intraclass correlation coefficients (ICC).

A McNemar test was used to test for differences in overall patient‐level detection rates between modalities.

The interreader and intrareader agreement was assessed using Cohen's *κ* for binary variables (overall positivity and miT) and using ICC with a two‐way random‐effects model for absolute agreement^27^ for nonbinary variables (miN and miM). The Landis and Koch classifications were used for the interpretations of *κ* and ICC values: 0.0–0.2, poor; 0.21–0.40, fair; 0.4–0.59, moderate; 0.6–0.79, substantial; 0.81–1.0, almost‐perfect reproducibility.^28^ The miM categories were converted to ordinal categorical scales: miM0 0, miM1a 1, miM1b 2, miM1c 3, for the ICC analysis. For the miM1b stage, the pattern of bone involvement was not considered in the analysis. For all statistical analyses, *P*‐values <0.05 were considered statistically significant.

## Results

Results from the detection rate analysis and intrareader agreement analysis were similar between reading teams and are presented for the primary reading team only. The results for the secondary reading team can be found as Data S1 and S2 in the Supplemental Material.

### Detection Rate Analysis

**FIGURE 2:**
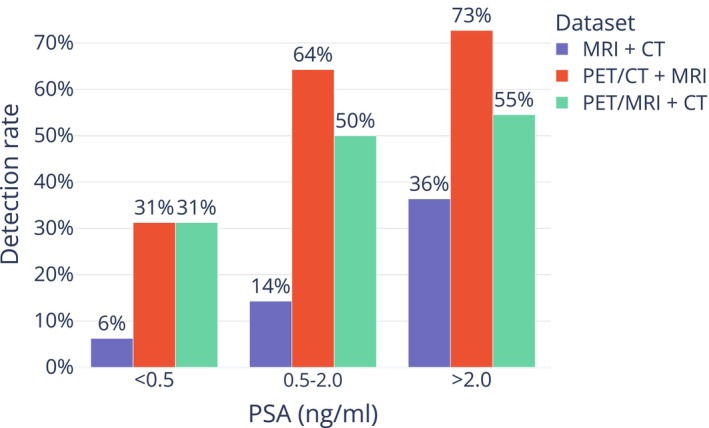
Overall detection rate for the primary reading team stratified by PSA level at time of imaging.

Detection rates for the primary reading team are presented in Table 3. The overall detection rates were significantly higher for the joint readings than for MRI + CT readings alone with 22 (54%) and 18 (44%) of patients with positive scans on PET/CT + MRI and PET/MRI + CT, respectively, compared with 7 (17%) on MR + CT. There was no significant difference in overall detection rates between PET/CT + MRI and PET/MRI + CT (*P* = 0.22). Furthermore, 17/18 (94%) of the patients that had positive scans in the PET/MRI + CT reading were also positive in the PET/CT + MRI reading. Overall detection rates for the primary reading team as a function of PSA level at imaging are presented in Fig. 2. The detection rate increased with increasing PSA for all datasets, with higher detection rates being observed in PET/CT + MRI and PET/MRI + CT datasets compared with MRI + CT.

**TABLE 3:** Region‐Based Detection Rate Analysis for All 41 Patients Presenting the Number of Patients for which Each Region was Positive for Each Dataset

	MRI + CT	PET/CT + MRI	PET/MRI + CT	MRI + CT ∩ PET/CT + MRI	MRI + CT ∩ PET/MRI + CT	PET/MRI + CT ∩ PET/CT + MRI
Positive scan	7 (17%)	22 (54%)	18 (44%)	7	6	17
Local recurrence	1 (2%)	6 (15%)	5 (12%)	1	1	3
Lymph nodes						
Pelvic	3 (7%)	12 (29%)	11 (27%)	2	2	10
Left Iliac	2 (5%)	5 (12%)	4 (10%)	1	1	4
Right Iliac	2 (5%)	5 (12%)	5 (12%)	1	1	5
Common Iliac	0 (0%)	5 (12%)	3 (7%)	0	0	3
Other	1 (2%)	2 (5%)	3 (7%)	1	1	2
Retroperitoneal	0 (0%)	3 (7%)	1 (2%)	0	0	1
Bone	4 (10%)	5 (12%)	6 (15%)	4	3	4
Pelvic	0 (0%)	2 (5%)	3 (7%)	0	0	1
Spine	4 (10%)	4 (10%)	3 (7%)	4	3	3
Other	0 (0%)	2 (5%)	1 (2%)	0	0	1

∩ indicates that the region is found to be positive for both datasets. The table shows data for the primary reading team. The left and right iliac regions contain the internal iliac nodes, external iliac nodes and obturator nodes.

CT = computed tomography; PET = positron emission tomography.

The addition of PET to MRI + CT led to more local recurrences being reported. Only 1 (2%) patient was positive for local recurrence in the MRI + CT reading, and 6 (15%) and 5 (12%) were considered positive for local recurrence in the PET/CT + MRI and PET/MRI + CT reading, respectively. However, a considerable discrepancy was observed between PET/CT + MRI and PET/MRI + CT, with only three patients being considered positive in both datasets.

Overall, more patients with positive findings and regions were found in the PET/CT + MRI reading than in the PET/MRI + CT reading. Moreover, PET/CT + MRI had additional positive regions compared with PET/MRI + CT for all major regions except for bone (eg, local recurrence, pelvic lymph nodes, and retroperitoneal lymph nodes). The largest differences were observed in the common iliac and retroperitoneal regions, where two additional patients had regions with positive lymph nodes on PET/CT + MRI compared with PET/MRI + CT. No visceral metastases were found in any of the readings.

### Intrareader and Interreader Agreement Analysis

The intrareader agreement for the primary reading team between MRI + CT, PET/CT + MRI, and PET/MRI + CT is presented in Table 4. For miT and miN stages there was poor agreement between MR + CT and either of the joint readings. For miM the agreement was moderate between PET/MRI + CT and MR + CT (ICC = 0.53, 95% confidence interval (CI) 0.28–0.72) and almost perfect between PET/CT + MRI and MRI + CT (ICC = 0.82, 95% CI 0.69–0.90). For overall positivity there was poor agreement between MRI + CT and either of the joint readings.

**TABLE 4:** Intrareader Variability Between Datasets for the Primary Reading Team Measured as Cohen's *κ* for miT and, Overall Positivity and ICC

Dataset		miT	miN	miM	Overall
MRI + CT	PET/CT + MRI	0.25 (−0.15–0.66)	0.25 (−0.03–0.50)	0.82 (0.69–0.90)	0.30 (0.10–0.50)
MRI + CT	PET/MRI + CT	0.31 (−0.16–0.77)	0.26 (−0.02–0.52)	0.53 (0.28–0.72)	0.31 (0.06–0.56)
PET/MRI + CT	PET/CT + MRI	0.48 (0.08–0.87)	0.93 (0.87–0.96)	0.68 (0.47–0.81)	0.71 (0.50–0.92)

Values are given with 95% CIs.

PET = positron emission tomography; CT = computed tomography.

Between PET/MRI + CT and PET/CT + MRI, there was moderate agreement for detection of local recurrences (*κ* = 0.48, 95% CI 0.08–0.87). In miN staging, there was almost perfect agreement (ICC = 0.93, 95% CI 0.87–0.96) and in miM staging there was substantial agreement (ICC = 0.68, 95% CI 0.47–0.81). We also found substantial agreement in overall patient positivity between PET/MRI + CT and PET/CT + MRI (*κ* = 0.71, 95% CI 0.50–0.92).

The interreader agreement between reading teams when assessing the same imaging datasets is shown in Table 5. For MRI + CT, poor agreement was found for overall positivity, miT, and miM staging, while almost perfect agreement was found in miN stages (ICC = 0.90, 95% CI 0.83–0.95).

**TABLE 5:** Interreader Variability Measured as Cohen's *κ* for miT and Overall Positivity, and ICC

Dataset	miT	miN	miM	Overall
MRI + CT	−0.02 (−0.06–0.01)	0.90 (0.83–0.95)	0.27 (−0.04–0.53)	0.36 (−0.02–0.74)
PET/CT + MRI	0.63 (0.26–1.00)	0.98 (0.96–0.99)	0.79 (0.63–0.88)	0.85 (0.70–1.01)
PET/MRI + CT	0.88 (0.64–1.11)	0.95 (0.91–0.97)	0.70 (0.50–0.83)	0.85 (0.69–1.01)

Values are given with 95% CIs.

CT = computed tomography; PET = positron emission tomography.

Additionally, PET/MRI + CT and PET/CT + MRI showed similar or better agreement than MR + CT across all miTNM sites. Almost perfect agreement was found between reading teams, for miN staging and for overall positivity for both PET/CT + MRI and PET/MRI + CT, and substantial agreement was obtained for miM staging. For miT staging, PET/CT + MRI showed substantial agreement (*κ* = 0.63, 95% CI 0.26–1.01) and PET/MRI + CT showed almost perfect agreement (*κ* = 0.88, 95% CI 0.64–1.11).

### Intention‐to‐Treat Analysis

The number of patients that had changes in clinical management between modalities for the primary reading team and between readers for the same modality is presented in Table 6. The addition of [^18^F]PSMA‐1007 PET to MRI + CT changed intended treatment in more than 40% of the patients, regardless of whether PET was acquired using PET/CT or PET/MRI.

**TABLE 6:** Number of Patients (*n*) With Changes in Intended Clinical Management Between Datasets for the Primary Reading Team (Intrareader), and Between Reading Teams for the Same Dataset (Interreader)

Intrareader			Interreader	
Dataset		*n*	Dataset	*n*
MRI + CT	PET/CT + MRI	20 (49%)	MRI + CT	13 (32%)
MRI + CT	PET/MRI + CT	18 (44%)	PET/CT + MRI	13 (32%)
PET/MRI + CT	PET/CT + MRI	8 (20%)	PET/MRI + CT	13 (32%)

CT = computed tomography; PET = positron emission tomography.

A substantially lower number of patients had a change in intended clinical management between PET/CT + MRI and PET/MRI + CT for the same readers (8 patients), as compared with the same PET modality between readers (PET/CT + MRI 13 patients, PET/MRI + CT 13 patients).

Of the 20 patients that had a change in clinical management between MRI + CT and PET/CT + MRI, PET/CT + MRI yielded more aggressive management for 10 patients and less aggressive management for 4 patients. For the remaining six patients, additional examinations were required before final treatment could be determined. Of the 18 patients that had a change in clinical management between MRI + CT and PET/MRI + CT, PET/MRI + CT yielded more aggressive management for 8 patients and less aggressive management for 3 patients. For the remaining seven patients, additional examinations were required before final treatment could be determined. Finally, between PET/CT + MRI and PET/MRI + CT, out of the eight patients that had a change in clinical management, PET/MRI + CT yielded more aggressive management for three patients and less aggressive management for three patients. For the remaining two, additional examinations were required before final treatment could be determined.

**FIGURE 3:**
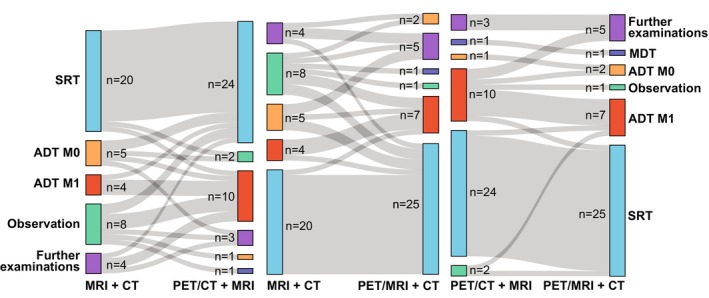
Sankey diagram showing the change in intended clinical management between datasets. The treatment categories are stratified into ADT with confirmed metastatic disease (ADT M1) and without confirmed metastatic disease (ADT M0), salvage radiotherapy (SRT), metastasis‐directed therapy (MDT), observation, and further examinations.

Figure 3 shows changes in clinical management categories between datasets based on the readings of the primary reading team. With the addition of PET_PET/CT_ or PET_PET/MRI_ to MRI + CT, more patients would have received active treatment as opposed to observation only.

Four out of the eight patients that had a change in intended treatment between PET/CT + MRI and PET/MRI + CT had the same miTNM stage in both datasets. Additionally, changes in intended treatment were generally more frequent in the patients that had received radiotherapy as primary or salvage treatment. A subgroup analysis can be found in the Data S3 in the Supplemental Material. Between oncologists, the intended treatment plan differed for 10 patients (24%) for MRI + CT, 10 patients (24%) for PET/CT + MRI, and 11 patients (27%) for PET/MRI + CT.

## Discussion

In this study, we examined the added value of [^18^F]PSMA‐1007 PET to MRI and CT for patients with BCR and residual disease after salvage treatment. In addition, we compared whether there was a difference in performance between PET from PET/CT and PET from PET/MRI. The addition of PET from either modality was found to increase overall detection rates, improve interreader agreement, and to change intended treatment in more than 40% of patients compared with MRI and CT alone. The performance of PET from either modality was found to be similar.

In accordance with similar head‐to‐head studies comparing [^68^Ga]Ga‐PSMA‐11 PET/CT and PET/MRI to MRI, our results showed that both PET/CT + MRI and PET/MRI + CT outperformed MRI and CT in localizing recurrent prostate cancer.^9^, ^12^ Comparing the detection rates of PET/MRI and PET/CT, our data showed a slightly larger difference than previously reported in similar studies.^9^, ^12^ This was also reflected in the region‐based analysis where PET/CT + MRI detected a higher number of lesions compared with PET/MRI + CT for all major regions except for bone. Other head‐to‐head studies found a similar number of discordant lesions by either modality^12^ or that PET/MRI discovered more lesions than PET/CT.^9^, ^11^

Time‐of‐flight capabilities which has been shown to increase the number of detected lesions in [^68^Ga]Ga‐PSMA‐11 examinations.^29^ In a study by Jentjens et al^9^ comparing the performance [^68^Ga]Ga‐PSMA‐11 PET/CT and PET/MRI for BCR only PET_PET/MRI_ was acquired using a scanner with time‐of‐flight capabilities. In our study, only the PET_PET/CT_ was acquired with a time‐of‐flight method, which may explain why more lesions were found for PET/CT compared with PET/MRI. Additionally, in our study, the imaging was optimized for PET_PET/CT_, which was performed 120 minutes after tracer injection and was within the range recommended in the recent joint European Association of Nuclear Medicine procedure guidelines and Society of Nuclear Medicine and Molecular Imaging procedure standard for prostate cancer imaging.^30^ For the majority of the patients, the PET_PET/MRI_ and pelvic PET_PET/MRI_ were acquired after PET_PET/CT_. An overview of the post‐injection times for the PET acquisitions can be found in the Data S4 in the Supplemental Material. The difference in acquisition times of PET_PET/CT_ and PET_PET/MRI_ may have contributed to some of the differences observed between joint readings.

The overall detection rates reported in this study are in agreement with those reported in a recent review of prospective studies on ^18^F‐ and ^68^Ga‐PSMA radioligands for patients with BCR and residual disease after salvage treatment.^31^ In the review, a pooled detection rate off 66.6% was found, with individual studies ranging from 31%^32^ to 85%.^33^ The PSA level is a known predictor of scan positivity.^34^ The relatively low median PSA level in our cohort may explain why our overall detection rates of 54% for PET/CT + MRI and 44% for PET/MRI + CT are toward the lower end of the range.^31^ In addition, some of the patients in our cohort had only pelvic PET examinations, which may have led to lesions outside the pelvic FOV being missed.

We observed a particularly large discrepancy in the detection of local recurrences between joint readings. Although only a single additional local recurrence was found in PET/CT + MRI compared with PET/MRI + CT, only three out of the eight local recurrences were found in both datasets. The remaining five local recurrences were unique to each dataset. In similar studies, Jentjens et al^9^ found local recurrences in the same 8 patients on PET/MRI and PET/CT in a total of 34 patients, and Guberina et al^11^ found 6 additional local recurrences for PET/MRI compared with PET/CT in a total of 93 patients. The additional detected local recurrences attributed to [^18^F]PSMA‐1007 PET in our study is in contrast to other studies where additional local recurrences were attributed to the MRI component.^11^, ^12^, ^35^, ^36^ However, these are all [^68^Ga]Ga‐PSMA‐11 studies where radiotracer accumulation in the bladder is more severe compared with [^18^F]PSMA‐1007, potentially masking uptake from local recurrences.^11^, ^12^, ^35^, ^36^ There are also differences in the study design, which makes it difficult to compare results.^12^, ^36^

37 out of the 41 patients (90%) included in this study had undergone radical prostatectomy. The sensitivity of multiparametric MRI for detection of local recurrence after radical prostatectomy was in a recent study reported to be 27.2%.^37^ In this study, only a single local recurrence was found on MRI + CT by either reading team. The large difference in local recurrences between MRI + CT and joint readings may be caused by false‐positive findings on [^18^F]PSMA‐1007 PET, or a lower sensitivity of multiparametric MRI.

The Intrareader agreement between PET/MRI + CT and PET/CT + MRI in our study ranged from moderate to almost perfect, where least agreement was observed for the miT stage, and the best for miN stage. These results are similar to those reported by Fendler et al^38^ and Demirci et al^39^ on [^68^Ga]Ga‐PSMA‐11 PET/CT.

Our results show that the addition of [^18^F]PSMA‐1007 PET to MRI and CT may lead to a change in intended clinical management in more than 40% of the patients. In a recent review, Mazrani et al^31^ reported an overall major change in clinical management in 42.7% of patients after PSMA PET/CT or PET/MRI, which is in line with our findings. In the review, a major change was defined as more than a change of site, radiation dose, or volume for the same modality/type of treatment.^31^ The change in intended treatment employed in our study is similar to Mazrani's definition of major change; however, a distinction was made between ADT with and without confirmed metastatic disease.^31^ In another recent systematic review, Pozdnyakov et al^8^ reported changes in clinical management after PSMA‐radioligand PET in 56.4% of patients. In the intention‐to‐treat analysis performed in our study, radiotherapy dose escalation to subvolumes based on imaging results was not taken into consideration, as was done by others.^4^, ^40^ If boost volumes were considered, we would likely have seen even more frequent changes in intended treatment with addition of PET to MRI and CT.

The difference in the assigned intended treatment plans between the urologic oncologists on the same dataset exceeded 20% for PET/MRI + CT, PET/CT + MRI, and MRI + CT. This may indicate that there is substantial interreader variability in assigned treatment even when the lesion findings and interpretations are the same.

Additionally, for four out of the eight patients where intended treatment differed between PET/MRI + CT and PET/CT + MRI for the primary reading team and oncologist, the miTNM score was the same. This may indicate that there is also likely some read‐repeat variation in the assigned treatments. These factors may have contributed to the interreader difference observed in intended treatment of almost one in three patients when comparing treatments plans made by the primary oncologist based on the same dataset between reading teams (e.g., comparing intended treatment based on PET/MRI + CT between the primary and secondary reading team).

### Limitations

There was no histopathological correlates and no systematic long‐term follow‐up data available to generate a composite reference standard. Therefore, we cannot distinguish true positive prostate cancer lesions from false‐positive findings. Also, as this is a retrospective intention‐to‐treat analysis where multiple treatment plans were assigned to each patient we cannot know the response to the proposed treatment. Furthermore, intended clinical management was determined by a urologic oncologist based on findings from radiologists and nuclear medicine physicians. Decisions made by a full multidisciplinary team, as is clinical practice, may have differed for some patients. Additionally, the suggested treatments would in a clinical setting have been discussed with the patient. The patient preference would in addition to other factors like individual life expectancy, health status, and frailty have impacted the final treatment decision. Another major limitation of this study was the small sample size. Owing to the small patient cohort, this study had limited power to uncover differences in detection rates between PET/MRI + CT and PET/CT + MRI. A larger study with a noninferiority design and histopathological correlates is needed to confirm that PET/MRI + CT is at least as accurate as PET/CT + MRI for localization of recurrent prostate cancer.

## Conclusion

In this study, we examined the added value of [^18^F]PSMA‐1007 PET to MRI and CT for patients with prostate cancer recurrence, and whether choice of PET modality, i.e., PET/MRI or PET/CT, had an impact on lesion detection, intrareader and interreader agreement, and subsequent clinical management. Our results may indicate similar overall detection rates for recurrent prostate cancer between PET/CT and PET/MRI when added to conventional imaging with MRI and CT. The addition of PET/CT or PET/MRI to conventional imaging with MRI and CT was found to increase overall positivity and interreader agreement in miTNM staging. A change in intended treatment could be observed in more than 40% of patients with the inclusion of [^18^F]PSMA‐1007 PET compared with using conventional imaging with CT and MRI.

## Funding Information

This work was supported by the Norwegian Cancer Society and Prostatakreftforeningen (Grant Number 215951), the Liaison Committee between the Central Norway Regional Health Authority and the Norwegian University of Science and Technology (Grant Numbers 90265300) and 180° N‐Norwegian Nuclear Medicine Consortium.

## Supporting information


**Data S1.** Supporting Information.

## References

[1] Han M , Partin AW , Pound CR , Epstein JI , Walsh PC . Long‐term biochemical disease‐free and cancer‐specific survival following anatomic radical retropubic prostatectomy. The 15‐year Johns Hopkins experience. Urol Clin North Am 2001;28:555‐565. 11590814 10.1016/s0094-0143(05)70163-4

[2] Widmark A , Klepp O , Solberg A , et al. Endocrine treatment, with or without radiotherapy, in locally advanced prostate cancer (SPCG‐7/SFUO‐3): An open randomised phase III trial. Lancet 2009;373:301‐308. 19091394 10.1016/S0140-6736(08)61815-2

[3] Cornford P , Bellmunt J , Bolla M , et al. EAU‐ESTRO‐SIOG guidelines on prostate cancer. Part II: Treatment of relapsing, metastatic, and castration‐resistant prostate cancer. Eur Urol 2017;71:630‐642. 27591931 10.1016/j.eururo.2016.08.002

[4] Farolfi A , Ceci F , Castellucci P , et al. 68Ga‐PSMA‐11 PET/CT in prostate cancer patients with biochemical recurrence after radical prostatectomy and PSA <0.5 ng/ml. Efficacy and impact on treatment strategy. Eur J Nucl Med Mol Imaging 2019;46:11‐19. 29905907 10.1007/s00259-018-4066-4

[5] Calais J , Fendler WP , Eiber M , et al. Impact of 68Ga‐PSMA‐11 PET/CT on the Management of Prostate Cancer Patients with biochemical recurrence. J Nucl Med 2018;59:434‐441. 29242398 10.2967/jnumed.117.202945 PMC5868499

[6] Roach PJ , Francis R , Emmett L , et al. The impact of 68Ga‐PSMA PET/CT on management intent in prostate cancer: Results of an Australian prospective multicenter study. J Nucl Med 2018;59:82‐88. 28646014 10.2967/jnumed.117.197160

[7] Sonni I , Eiber M , Fendler WP , et al. Impact of 68Ga‐PSMA‐11 PET/CT on staging and Management of Prostate Cancer Patients in various clinical settings: A prospective single‐center study. J Nucl Med 2020;61:1153‐1160. 31924715 10.2967/jnumed.119.237602 PMC7413232

[8] Pozdnyakov A , Kulanthaivelu R , Bauman G , Ortega C , Veit‐Haibach P , Metser U . The impact of PSMA PET on the treatment and outcomes of men with biochemical recurrence of prostate cancer: A systematic review and meta‐analysis. Prostate Cancer Prostatic Dis 2022;26:240‐248. 35440642 10.1038/s41391-022-00544-3

[9] Jentjens S , Mai C , Ahmadi Bidakhvidi N , et al. Prospective comparison of simultaneous [68Ga]Ga‐PSMA‐11 PET/MR versus PET/CT in patients with biochemically recurrent prostate cancer. Eur Radiol 2022;32:901‐911. 34374802 10.1007/s00330-021-08140-0

[10] Freitag MT , Radtke JP , Hadaschik BA , et al. Comparison of hybrid 68Ga‐PSMA PET/MRI and 68Ga‐PSMA PET/CT in the evaluation of lymph node and bone metastases of prostate cancer. Eur J Nucl Med Mol Imaging 2016;43:70‐83. 26508290 10.1007/s00259-015-3206-3

[11] Guberina N , Hetkamp P , Ruebben H , et al. Whole‐body integrated [68Ga]PSMA‐11‐PET/MR imaging in patients with recurrent prostate cancer: Comparison with whole‐body PET/CT as the standard of reference. Mol Imaging Biol 2019;22:788‐796. 10.1007/s11307-019-01424-4 31482413

[12] Glemser PA , Rotkopf LT , Ziener CH , et al. Hybrid imaging with [68Ga]PSMA‐11 PET‐CT and PET‐MRI in biochemically recurrent prostate cancer. Cancer Imaging 2022;22:53. 36138437 10.1186/s40644-022-00489-9 PMC9502876

[13] Liu T , Wang S , Liu H , et al. Detection of vertebral metastases: A meta‐analysis comparing MRI, CT, PET, BS and BS with SPECT. J Cancer Res Clin Oncol 2017;143:457‐465. 27752772 10.1007/s00432-016-2288-z PMC11819241

[14] Giesel FL , Hadaschik B , Cardinale J , et al. F‐18 labelled PSMA‐1007: Biodistribution, radiation dosimetry and histopathological validation of tumor lesions in prostate cancer patients. Eur J Nucl Med Mol Imaging 2017;44:678‐688. 27889802 10.1007/s00259-016-3573-4 PMC5323462

[15] Arnfield EG , Thomas PA , Roberts MJ , et al. Clinical insignificance of [18F]PSMA‐1007 avid non‐specific bone lesions: A retrospective evaluation. Eur J Nucl Med Mol Imaging 2021;48:4495‐4507. 34136957 10.1007/s00259-021-05456-3

[16] EAU Guidelines Office . Edition Presented at the EAU annual congress Amsterdam 2022. Arhen, Netherlands: EAU Guidelines Office; 2022.

[17] Helsedirektoratet . Nasjonalt handlings program med retningslinjer for diagnostikk, behandling og oppfølging av prostatakreft. 11th ed. Oslo: Helsedirektoratet; 2021.

[18] Ollinger JM . Model‐based scatter correction for fully 3D PET. Phys Med Biol 1996;41:153‐176. 8685253 10.1088/0031-9155/41/1/012

[19] Paulus DH , Quick HH , Geppert C , et al. Whole‐body PET/MR imaging: Quantitative evaluation of a novel model‐based MR attenuation correction method including bone. J Nucl Med 2015;56:1061‐1066. 26025957 10.2967/jnumed.115.156000 PMC4894503

[20] Delso G , Furst S , Jakoby B , et al. Performance measurements of the Siemens mMR integrated whole‐body PET/MR scanner. J Nucl Med 2011;52:1914‐1922. 22080447 10.2967/jnumed.111.092726

[21] Lowekamp B , Chen D , Ibanez L , Blezek D . The design of SimpleITK. Front Neuroinform 2013;7. 10.3389/fninf.2013.00045 PMC3874546 24416015

[22] Yaniv Z , Lowekamp BC , Johnson HJ , Beare R . SimpleITK image‐analysis notebooks: A collaborative environment for education and reproducible research. J Digit Imaging 2018;31:290‐303. 29181613 10.1007/s10278-017-0037-8 PMC5959828

[23] Ceci F , Oprea‐Lager DE , Emmett L , et al. E‐PSMA: The EANM standardized reporting guidelines v1.0 for PSMA‐PET. Eur J Nucl Med Mol Imaging 2021;48:1626‐1638. 33604691 10.1007/s00259-021-05245-y PMC8113168

[24] Eiber M , Herrmann K , Calais J , et al. Prostate cancer molecular imaging standardized evaluation (PROMISE): Proposed miTNM classification for the interpretation of PSMA‐ligand PET/CT. J Nucl Med 2018;59:469‐478. 29123012 10.2967/jnumed.117.198119

[25] Seabold S , Perktold J . Statsmodels: Econometric and statistical modeling with python. In 9th Python Science Conference. 2010.

[26] Vallat R . Pingouin: Statistics in python. J Open Source Softw 2018;3:1026.

[27] Koo TK , Li MY . A guideline of selecting and reporting Intraclass correlation coefficients for reliability research. J Chiropr Med 2016;15:155‐163. 27330520 10.1016/j.jcm.2016.02.012 PMC4913118

[28] Landis JR , Koch GG . The measurement of observer agreement for categorical data. Biometrics 1977;33:159‐174. 843571

[29] Krohn T , Birmes A , Winz OH , et al. The reconstruction algorithm used for [68Ga]PSMA‐HBED‐CC PET/CT reconstruction significantly influences the number of detected lymph node metastases and coeliac ganglia. Eur J Nucl Med Mol Imaging 2017;44:662‐669. 27900518 10.1007/s00259-016-3571-6

[30] Fendler WP , Eiber M , Beheshti M , et al. PSMA PET/CT: Joint EANM procedure guideline/SNMMI procedure standard for prostate cancer imaging 2.0. Eur J Nucl Med Mol Imaging 2023;50:1466‐1486. 36604326 10.1007/s00259-022-06089-w PMC10027805

[31] Mazrani W , Cook GJR , Bomanji J . Role of 68Ga and 18F PSMA PET/CT and PET/MRI in biochemical recurrence of prostate cancer: A systematic review of prospective studies. Nucl Med Commun 2022;43:631‐637. 35438666 10.1097/MNM.0000000000001557

[32] Afaq A , Payne H , Davda R , et al. A phase II, open‐label study to assess safety and management change using 68Ga‐THP PSMA PET/CT in patients with high‐risk primary prostate cancer or biochemical recurrence after radical treatment: The PRONOUNCED study. J Nucl Med 2021;62:1727‐1734. 33741648 10.2967/jnumed.120.257527 PMC8612191

[33] Song H , Harrison C , Duan H , Guja K , Hatami N , Franc BL . Prospective evaluation of 18F‐DCFPyL PET/CT in biochemically recurrent prostate cancer in an academic center: A focus on disease localization and changes in management. J Nucl Med 2020;61:546‐551. 31628216 10.2967/jnumed.119.231654

[34] Ahmadi Bidakhvidi N , Laenen A , Jentjens S , et al. Parameters predicting [18F]PSMA‐1007 scan positivity and type and number of detected lesions in patients with biochemical recurrence of prostate cancer. EJNMMI Res 2021;11:41. 33929626 10.1186/s13550-021-00783-w PMC8087750

[35] Joshi A , Roberts MJ , Perera M , et al. The clinical efficacy of PSMA PET/MRI in biochemically recurrent prostate cancer compared with standard of care imaging modalities and confirmatory histopathology: Results of a single‐centre, prospective clinical trial. Clin Exp Metastasis 2020;37:551‐560. 32519046 10.1007/s10585-020-10043-1

[36] Freitag MT , Radtke JP , Afshar‐Oromieh A , et al. Local recurrence of prostate cancer after radical prostatectomy is at risk to be missed in 68Ga‐PSMA‐11‐PET of PET/CT and PET/MRI: Comparison with mpMRI integrated in simultaneous PET/MRI. Eur J Nucl Med Mol Imaging 2017;44:776‐787. 27988802 10.1007/s00259-016-3594-z

[37] Kim M , Hwang SI , Ahn H , et al. Diagnostic yield of multiparametric MRI for local recurrence at biochemical recurrence after radical prostatectomy. Prostate Int 2022;10:135‐141. 36225284 10.1016/j.prnil.2022.05.001 PMC9520418

[38] Fendler WP , Calais J , Eiber M , et al. Assessment of 68Ga‐PSMA‐11 PET accuracy in localizing recurrent prostate cancer: A prospective single‐arm clinical trial. JAMA Oncol 2019;5:856‐863. 30920593 10.1001/jamaoncol.2019.0096 PMC6567829

[39] Demirci E , Akyel R , Caner B , et al. Interobserver and intraobserver agreement on prostate‐specific membrane antigen PET/CT images according to the miTNM and PSMA‐RADS criteria. Nucl Med Commun 2020; 41:759‐767. 32453205 10.1097/MNM.0000000000001219

[40] Müller J , Ferraro DA , Muehlematter UJ , et al. Clinical impact of 68Ga‐PSMA‐11 PET on patient management and outcome, including all patients referred for an increase in PSA level during the first year after its clinical introduction. Eur J Nucl Med Mol Imaging 2019;46:889‐900. 30488099 10.1007/s00259-018-4203-0

