# Supplementary material for: Added Value of [18F]PSMA‐1007 PET/CT and PET/MRI in Patients With Biochemically Recurrent Prostate Cancer: Impact on Detection Rates and Clinical Management
Source: J Magn Reson Imaging. 2024 Apr 28;61(1):466–77. doi: 10.1002/jmri.29386 (PMC11645485; doi:10.1002/jmri.29386)
Supplement: Supplementary file 1 — Data S1. Supporting Information. [file JMRI-61-466-s001.pdf]

Supplementary material

S.1 Detection rate analysis

Table 1: Region-based detection rate analysis presenting the number of patients for which each region was positive for each dataset. ∩ indicates that the region is found to be positive for both datasets. The table shows data for the secondary reading team. The left and right iliac regions contain the internal iliac nodes, external iliac nodes and obturator nodes.

Region	MRI + CT	PET/CT + MRI	PET/MRI + CT	MRI + CT ∩ PET/CT + MRI	MRI + CT ∩ PET/MRI + CT	PET/MRI + CT ∩ PET/CT + MRI
Positive scan (N = 41)	6 (15%)	19 (46%)	17 (41%)	6	6	17
Local Recurrence	1 (2%)	4 (10%)	4 (10%)	0	0	4
Lymph nodes						
Pelvic	4 (10%)	12 (29%)	10 (24%)	3	3	10
Left Iliac	4 (10%)	4 (10%)	5 (12%)	1	3	3
Right Iliac	1 (2%)	6 (15%)	6 (15%)	1	1	6
Common Iliac	2 (5%)	7 (17%)	6 (15%)	1	1	6
Other	1 (2%)	4 (10%)	2 (5%)	1	1	2
Retroperitoneal	2 (5%)	3 (7%)	2 (5%)	1	1	2
Bone	2 (5%)	5 (12%)	5 (12%)	2	2	5
Pelvic	2 (5%)	3 (7%)	3 (7%)	1	1	3
Spine	1 (2%)	3 (7%)	3 (7%)	1	1	3
Other	0 (0%)	2 (5%)	2 (5%)	0	0	2

S.2 Intra- and interreader variability

Table 2: Intrareader variability for the secondary reading team. The values are Cohen's κ for miT and overall positivity, and ICC. Values are given with 95% confidence intervals. The coloring represents different criteria for interpreting the κ and ICCs according to Landis and Koch [1]. Green is almost perfect, yellow is substantial, orange is moderate and red is poor reproducibility.

Intrareader Dataset		miT	miN	miM	Overall
MRI + CT	PET/CT + MRI	-0.04 (-0.10-0.02)	0.33 (0.04-0.57)	0.47 (0.20-0.68)	0.33 (0.11-0.55)
MRI + CT	PET/MRI + CT	-0.04 (-0.11-0.02)	0.42 (0.14-0.64)	0.49 (0.23-0.69)	0.39 (0.15-0.63)
PET/MRI + CT	PET/CT + MRI	0.84 (0.55-1.14)	0.86 (0.75-0.92)	0.97 (0.95-0.99)	0.90 (0.77-1.03)

S.3 Change in clinical management

Table 3: Change in intended clinical management between readers and datasets. n is the number of patients for which a change in intended management occurs. RT and No RT are the patients that had and had not previously received radiotherapy as initial treatment or salvage treatment. The percentage number reflects the proportion of patients within the given subgroup with a change in intended clinical management. R1 and R2 signify that the treatment is set based on data from the primary and secondary reading teams respectively.

Reader		Dataset				
R1	R2	M1	M2	n	n (RT)	n (no RT)
Between datasets						
R1	R1	PET/CT + MRI	MRI + CT	20 (48.8%)	14 (82.4%)	6 (25.0%)
R1	R1	PET/MRI + CT	MRI + CT	18 (43.9%)	13 (76.5%)	5 (20.8%)
R1	R1	PET/MRI + CT	PET/CT + MRI	7 (17.1%)	3 (17.6%)	4 (16.7%)
R2	R2	PET/CT + MRI	MRI + CT	16 (39.0%)	10 (58.8%)	6 (25.0%)
R2	R2	PET/MRI + CT	MRI + CT	16 (39.0%)	12 (70.6%)	4 (16.7%)
R2	R2	PET/MRI + CT	PET/CT + MRI	11 (26.8%)	9 (52.9%)	2 (8.3%)
Between reading teams						
R1	R2	MRI + CT	MRI + CT	13 (31.7%)	7 (41.2%)	6 (25.0%)
R1	R2	PET/CT + MRI	PET/CT + MRI	13 (31.7%)	10 (58.8%)	3 (12.5%)
R1	R2	PET/MRI + CT	PET/MRI + CT	12 (29.3%)	9 (52.9%)	3 (12.5%)
Between oncologists						
R1	R1	MRI + CT	MRI + CT	10 (24.4%)	8 (47.1%)	2 (8.3%)
R1	R1	PET/CT + MRI	PET/CT + MRI	10 (24.4%)	8 (47.1%)	2 (8.3%)
R1	R1	PET/MRI + CT	PET/MRI + CT	12 (29.3%)	9 (52.9%)	3 (12.5%)

S.4 Post-injection times

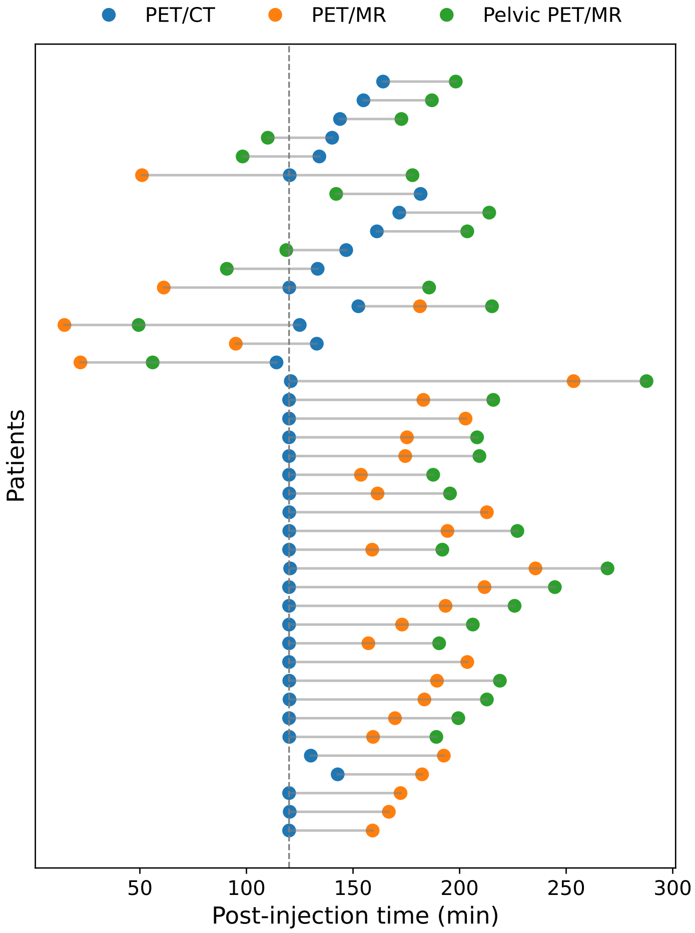

Figure 1: Post-injection times for the patients in the dataset. Each line corresponds to one patient, and each point represents one PET acquisition.

References

1. Landis, J. R. & Koch, G. G. The Measurement of Observer Agreement for Categorical Data. *Biometrics* **33,** 159–174 (1977).
